# The Effect of Molding Temperature on the Mechanical and Tribological Properties of Modified Phenolic Resin-Based Friction Materials

**DOI:** 10.1021/acsomega.5c00960

**Published:** 2025-05-16

**Authors:** Xiao Zhao, Wei Sun, Siyi Chen, Libei Zhan, Yuting Zhou, Jinwei He, Daocheng Luan, Zhihua Hu, Zhengyun Wang

**Affiliations:** College of Materials Science and Engineering & Key Laboratory of Materials and Surface Technology of Ministry of Education & Key Laboratory of Fluid and Power Machinery of Ministry of Education, 12598 Xihua University, Chengdu 610039, Sichuan, China

## Abstract

In order to investigate the influence of molding temperature on the mechanical and tribological properties of modified phenolic resin-based friction materials, six kinds of friction materials (FMs) with different molding temperatures were prepared by a hot-pressing process using melamine-modified phenolic resin (MPR) and boron-modified phenolic resin (BPR) as binders. The experimental results show that the density change of the friction materials is very small. The hardness, compressive strength, and compressive modulus decrease with the increase of molding temperature, and the impact strength decreases with the increase of molding temperature. The coefficient of friction (COF) of the melamine-modified phenolic resin-based friction material with a molding temperature of 160 °C is higher, and the stability is the largest. The COF of the boron-modified phenolic resin-based friction material decreases with the increase of the molding temperature, and the COF of each sample has a large difference. When the molding temperature is 160 °C, the COF of the samples is stable between 0.409 and 0.462 at different initial braking speeds. Relatively speaking, the sample with a hot-pressing temperature of 160 °C has better comprehensive properties, the wear mechanism is mainly abrasive wear and adhesive wear, and the large-area continuous friction film can stabilize the COF.

## Graphical abstract

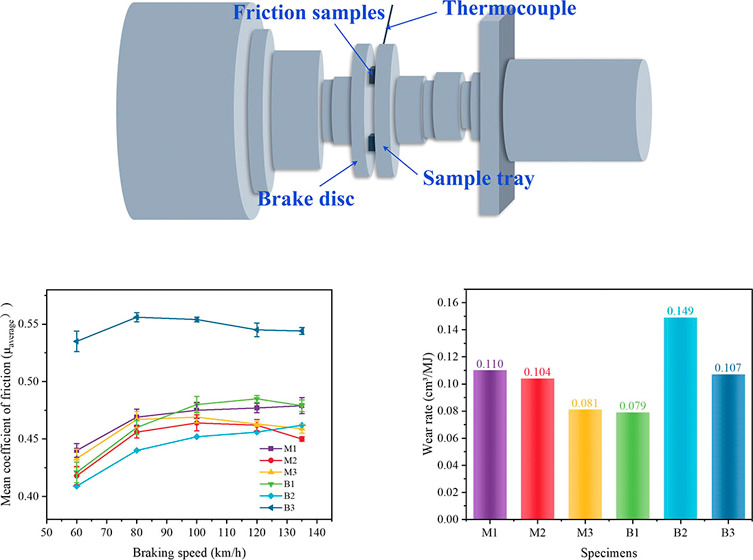

## 1 Introduction

As an important part of the braking system, friction materials directly affect the safety, stability, and comfort of the vehicle.^1^ So, friction materials are required to have good mechanical strength and physical properties, suitable and stable friction coefficient, and good wear resistance.^2,3^ The brake pads used at present are mainly nonasbestos-type friction materials, which can not only meet the braking requirements but also have the advantages of low preparation cost, simple preparation process, and strong designability.^4,5^ Nonasbestos-type organic friction materials are mainly composed of binders, reinforcing fibers, frictional property regulators, and fillers, among which the thermosetting phenolic resin is the most widely used binder.^6,7^

With the development of vehicle manufacturing technology, the performance requirements of friction materials are getting higher and higher, and an ordinary phenolic resin cannot meet the needs of use.^8–12^ When the braking temperature of an ordinary phenolic resin exceeds 200 °C, the methylene and phenolic hydroxyl groups on its main chain are easily oxidized, resulting in poor heat resistance.^13,14^ In addition, after curing, the phenolic resin benzene ring is only connected by methylene, and its high hardness and modulus make the friction material brittle, leading to poor performance and high braking noise.^15,16^ The poor heat resistance of the resin will lead to thermal decay, thermal cracking, and thermal expansion of friction materials. In order to solve the above problems and further improve the properties of friction materials, researchers adopted many methods to modify them, which can generally be divided into chemical modification and physical modification. Chemical modification involves introducing flexible chains and heat-resistant groups into the resin molecular chains through chemical reactions or regulating the cross-linking density via cross-linking reactions.^17–26^ Physical modification, on the other hand, employs blending methods to form an interpenetrating network structure between a phenolic resin and inorganic nanoparticles, rubber, etc., thereby enhancing its performance.^27^ Using Tung oil, vegetable oil (cashew shell oil, linseed oil, etc.), boron-containing compounds, nanoparticles, and polymer materials to modify a phenolic resin improves its toughness and heat resistance. Cashew shell oil can significantly improve the thermal decomposition temperature of the phenolic resin matrix, restrain the thermal decay phenomenon of friction materials, and improve the high-temperature friction properties.^28–30^ The heat resistance and toughness of a phenolic resin modified by NBR (nitrile butadiene rubber) were obviously improved. The polymer material can significantly increase the thermal decomposition temperature of the phenolic resin. An epoxy resin can significantly improve the adhesion, toughness, and corrosion resistance of a phenolic resin.^31,32^

In addition, adding boron to a phenolic resin forms “C–O–B” and “C–O → B” coordination bonds in phenolic macromolecular chains, and the bond energy increases. At the same time, boron phenolic resin easily forms a honeycomb structure of the boron carbide insulation layer at high temperature, which can protect the internal structure and prevent heat from spreading to the inside of the material. The friction material exhibits good thermal stability. At the same time, the B–O bond has good flexibility, and the toughness of the modified phenolic resin can also be improved.^33–36^ Some researchers found that MPF has a melamine ring, which increases the thermal decomposition temperature of the phenolic resin and improves the heat resistance.^37^ Bülent Öztürk et al.^38^ studied the frictional properties of friction materials using a pure phenolic resin, cashew shell oil-modified phenolic resin, and melamine resin as binders and found that the friction coefficient and wear rate of friction materials using MPR (melamine-modified phenolic resin) as a binder were the lowest. Peng Cai et al.^15^ simultaneously used NBR (nitrile rubber)-modified phenolic resin and BPR (boron-modified phenolic resin) as bonding agents for friction materials to prepare resin-based friction materials and found that the synergistic effect of the two modified resins made the friction materials not only have higher toughness but also have higher heat resistance. Therefore, in this study, MPR and BPR and a certain amount of nitrile rubber were used as bonding agents for friction materials.

The composition of a resin-based friction material is not only a binder and a reinforcement fiber but also a friction property regulator and a filler. Graphite and molybdenum disulfide have a layered structure, which can fracture along the molecular layer to produce a sliding surface during friction to reduce the friction of the sliding interface.^39,40^ Copper powder, as an isotropic crystalline substance with low Mohs hardness, has a good friction effect. In addition, copper powder has good thermal conductivity, which can reduce the temperature of the friction surface and slow the thermal decay of the friction material. The Mohs hardness of alumina is 7, and the higher hardness makes it scratch with the dual surface during the friction process, providing the main mechanical meshing force and increasing the friction coefficient.^31^ Therefore, these materials are commonly used as friction performance regulators in friction materials.

The properties of friction materials are affected not only by raw materials and their formulations and structural design but also by their preparation process. Phenolic resin-based friction materials are mainly prepared by one-time hot pressing. This method is to put the compressed material into the mold, press and cure under heating and pressure, and maintain a certain time to form a product.^41,42^ In this process, the phenolic resin in the pressed plastic will undergo three states under heating: viscous fluid state, gelatinization state, and hardening state. The phenolic resin in the viscous fluid state and gelatinization state is in a flow softening state. At this time, the resin macromolecules have a linear structure and a partial branched-chain structure, which makes the pressed plastic. Whether the pressed plastic can fill the mold cavity uniformly depends on the fluidity of the viscous fluid resin. After this stage, the phenolic resin enters a hardened state, and the molecular structure further forms a cross-linked structure, which combines the components together in the form of a bonded film.^37,43–45^ In the whole process of hot pressing, the molding temperature, pressure, and time all affect the performance of the product, but the molding temperature is the most critical factor affecting the flow and curing speed of the phenolic resin, the higher the molding temperature, the greater the flow speed and curing speed of the resin.^46,47^ Too low of a molding temperature will reduce production efficiency and affect the appearance and quality of products. The molding temperature is too high, and the resin hardening speed is too fast, which will reduce the fluidity of the pressed plastic relatively, resulting in loose and uneven texture of the product, and the mechanical strength does not meet the requirements. In addition, the high temperature will also make the water and volatiles inside the pressed plastic difficult to escape, and the product is hot pressed to complete the release with a great steam pressure to break through the surface of the product, resulting in bubbles, swelling, and cracks on the surface of the product.^48^ Reasonable and appropriate hot-pressing temperature not only helps to improve the fluidity of the resin, improve the interface combination between the matrix and the components, so that the friction material can have good mechanical properties and friction and wear properties to withstand the thermal stress and mechanical force generated during the braking process, but also has important significance for improving production efficiency and reducing costs.^49–51^

Therefore, in this study, BPR and MPR were used as the matrix of friction materials to prepare friction materials with different molding temperatures, and the influence of molding temperature on the mechanical tribological properties of the two modified phenolic resin-based friction materials was studied, which provided a certain theoretical basis for subsequent experimental research on resin-based friction materials and optimization of the molding process.

## 2 Experimental Section

### 2.1 Preparation and Formulation of the Sample

The formulation of friction material samples in this study is shown in Table 1. The amounts of reinforcing fiber, friction modifier, and filler are fixed. And two kinds of modified phenolic resin friction materials were prepared by mixing MPR and BPR with nitrile butadiene rubber, respectively. Among the other components, silica, high-temperature wear agents, graphite, copper powder, alumina, and molybdenum sulfide were used as the friction modifier components, and Barite was used as the filler component.

**Table 1:** Formulation of Friction Materials

composition	ratio (wt %)
modified phenolic resin	12
nitrile rubber	12
steel fiber	14
basalt fiber	14
other components	48

The friction materials were manufactured by using the a dry hot-press molding technique. All ingredients were mixed by a HLO-400A mixer with a rotational speed of 2500 rpm. First, the reinforced fibers were mixed for 3 min. After that, the friction modifiers and fillers were added to the mixture and blended for 2 min, and finally binders were added and mixed for 3 min. The mold used for the experiment is shown in Figure 1B. The mixed powders were put into the oven to dry at 60 °C for 4 h, and then the dry raw materials were hot-pressed at 155 °C, 160, and 165 °C, respectively, at 30 MPa for 15 min with a plate hot-pressing equipment, resulting in a sample size of 52 mm × 52 mm × 20 mm, and the sample is shown in Figure 1A. The specific hot-pressing process is shown in Table 2, and the numbers are M1, M2, M3, B1, B2, and B3, where M and B stand for MPR-based friction materials and BPR-based friction materials, respectively. The samples were placed in a high-temperature electric furnace and heated up from room temperature for 1.5 h to 100 °C for 0.5 h, 0.5 h to 140 °C for 0.5 h, and 0.5 h to 160 °C for 0.5 h and then cooled down with the furnace. For the subsequent tests, the heat-treated samples were machined and cut to predetermined dimensions.

**Figure 1:**
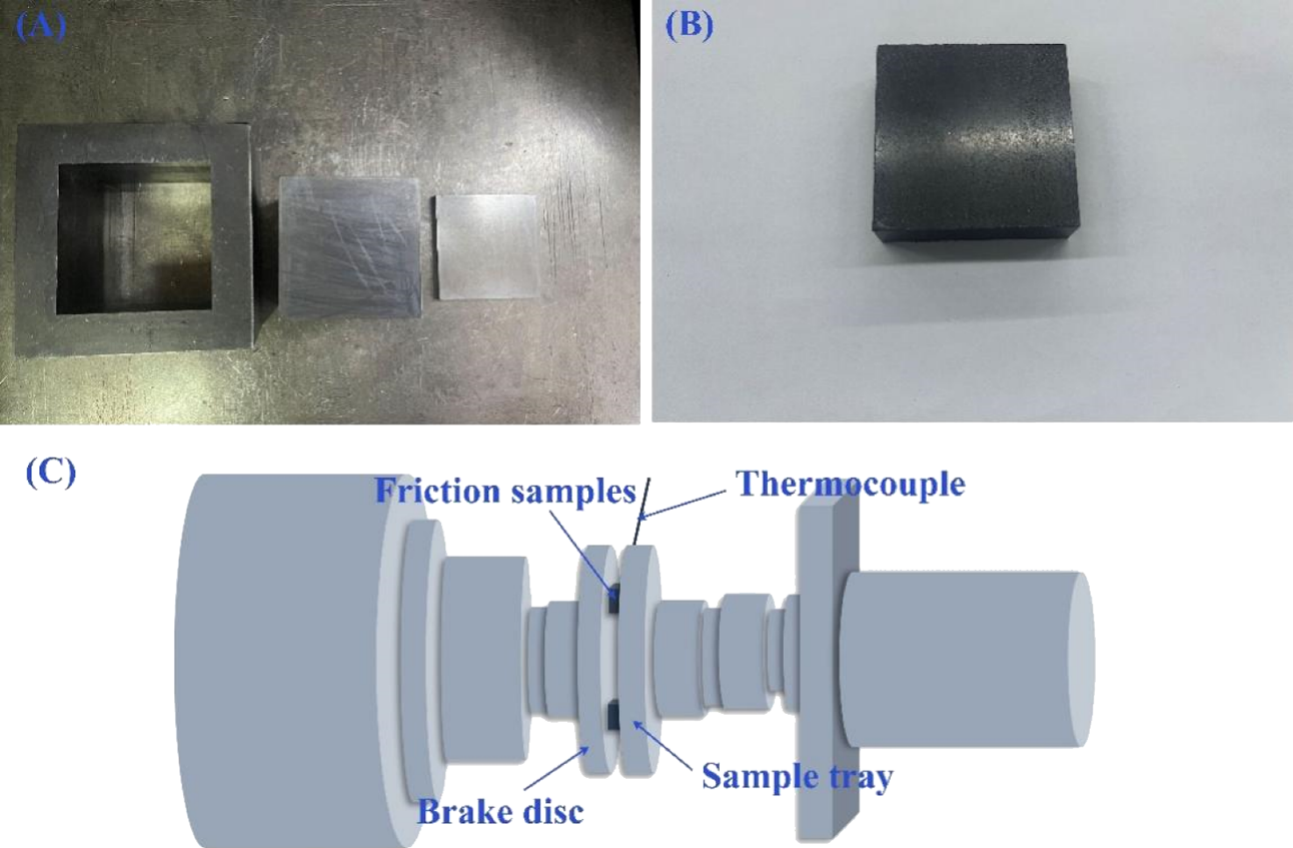
(A) Mold used for the experiment, (B) samples, and (C) diagram of the friction test device.

**Table 2:** Hot-Pressing Process

specimens	hot-pressing pressure (MPa)	hot-pressing temperature (°C)	hot-pressing time (min)
M1	30	155	15
M2	30	160	15
M3	30	165	15
B1	30	155	15
B2	30	160	15
B3	30	165	15

### 2.2 Performance Characterization

#### 2.2.1 Mechanical and Thermal Performance Test

The thermogravimetric analysis of the two phenolic resins used in the experiment was carried out with a Mettler Toledo TGA2 thermogravimetric analyzer under a nitrogen atmosphere, the heating rate was 10 °C/min, and the test temperature was from 25 to 800 °C. The density of the specimens was determined using the Archimedes drainage weighing method. The compression strength and modulus of the specimens were measured using a HD-B607-S high-temperature material universal testing equipment. The impact strength of the specimens was measured by using the GT-7045-MDL impact tester, which has an impact energy of 1 J and a basic velocity of 2.9 m/s. The hardness (R scale) of the specimens was determined by using an XHR-150 Rockwell hardness tester.

#### 2.2.2 Frictional Test

The friction test was carried out by an MM3000 small-scale analogue braking inertia testing machine, and its structure diagram is shown in Figure 1C. The brake pressure and test inertia of the testing machine are 0.4 MPa and 1.11–1.57 kg·m^2^, respectively. The coupling disc is made of aluminum. The test friction sample size is 15 mm × 15 mm × 20 mm, and there are three in total. A sample was drilled on its side 4 mm away from the friction surface to record the temperature change of the sample during friction. When the initial temperature of the sample does not exceed 60 °C and the braking speed is 80 km/h, the contact area between the friction surface of the sample and the brake disc is greater than 80%, and then the test is started. The braking speed is 60, 80, 100, 120, and 135 km/h, respectively, and the braking is repeated four times at each speed. The friction coefficient was recorded automatically by the device, and the wear rate *L* (cm^3^/MJ) is calculated as follows.

$$L=\frac{w_{1}-w_{2}}{\rho A}$$

where *w*_1_ is the mass of the sample before the friction test, g; *w*_2_ is the mass of the sample after the friction test, g; ρ is the density of the sample, g/cm^3^; and *A* is the total braking work throughout the test, J.

#### 2.2.3 Characterization of the Microscopic Structure

After friction and wear testing, the S-3400N scanning electron microscope (SEM) in conjunction with energy-dispersive X-ray spectroscopy (EDS) was utilized for analyzing the microscopic morphology of the specimens.

## 3 Result and Discussion

### 3.1 Thermal Behavior

When the organic binder is heated to a certain temperature, it softens into a viscous flow state and has fluidity so that it is evenly distributed in the matrix material. Subsequently, through further heating and pressure, the curing effect of the resin promotes the fiber and filler in the friction material to combine with each other and finally forms a dense texture and has a certain strength, which meets the requirements of the use of friction materials. Figure 2a depicts the TG and DTG curves of MPR. According to the TG curves, the thermogravimetric curve of MPR is used in the test. The first obvious thermal weight loss occurred near 155–165 °C, which is because the curing of the thermosetting resin is an exothermic reaction, and the curing reaction of MPR occurred in this range. The second sharp thermogravimetry is mainly due to the decomposition failure of MPR at about 526 °C. Figure 2b shows the TG and DTG curves of BPR. BPR used in the test showed an obvious mass loss at 155–165 °C and a distinct decomposition failure at 544 °C. According to the thermogravimetric curves of the two modified resins, the pressing temperatures of the process parameters were selected as 155 °C, 160, and 165 °C, respectively.

**Figure 2:**
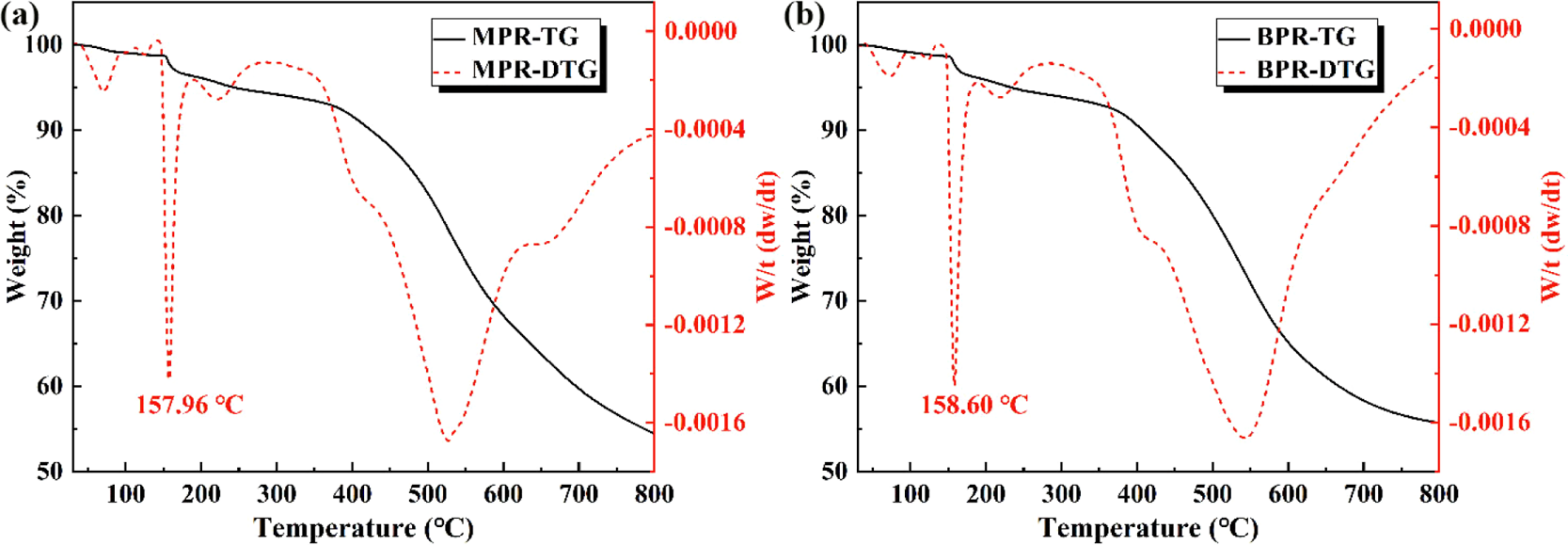
Thermogravimetric analysis of the modified phenolic resin, (a) MPR and (b) BPR.

### 3.2 Physical and Mechanical Properties

The physical and mechanical properties with differing phenolic resins and hot-pressing temperatures are compiled in Table 3. The density of the two modified phenolic resin-based friction materials fluctuated with the increase of the hot-pressing temperature, and the general fluctuation was not large. In general, the density of BPR-based friction materials is higher than that of MPR-based friction materials. The hardness of the two modified phenolic resin-based friction materials has the same change trend and decreases with the increase of the hot-pressing temperature. The curing degree of the phenolic resin increases with the increase of temperature, which makes the bonding effect more significant. However, if the curing temperature is too high than its reasonable curing temperature, it will make the binder gradually fail, and the combination between the components is no longer tight, resulting in a decrease in the hardness of the material.

**Table 3:** Physical and Mechanical Properties of Different Samples

specimens	density (g/cm^3^)	hardness (HRR)	impact strength (kJ/m^3^)	compressive strength (MPa)	compression modulus (MPa)
M1	2.295 ± 0.026	66.9 ± 4.7	3.705 ± 0.016	42.328 ± 1.969	379 ± 26
M2	2.274 ± 0.016	64.1 ± 3.9	4.095 ± 0.046	38.366 ± 1.764	329 ± 28
M3	2.303 ± 0.007	56.2 ± 4.2	3.960 ± 0.083	35.488 ± 2.936	299 ± 15
B1	2.271 ± 0.024	67.9 ± 1.9	3.460 ± 0.040	42.267 ± 0.659	419 ± 19
B2	2.326 ± 0.014	64.9 ± 0.4	3.747 ± 0.074	42.671 ± 0.830	359 ± 11
B3	2.315 ± 0.011	38.1 ± 1.4	4.193 ± 0.086	35.353 ± 0.757	325 ± 8

Friction materials will be subjected to a certain impact force during braking, so it is necessary to test the impact strength of the materials. If the impact strength of the material is too low, it is easy to appear brittle fracture during braking, which affects the further performance of the material. Table 3 shows that the impact strength of the two resin-based friction materials is the lowest when the hot-pressing temperature is 155 °C, which is 3.705 kJ/m^3^ and 3.460 kJ/m^3^, respectively. This is because when the hot-pressing temperature is 155 °C, the temperature is low, the hardening rate is slow, and the curing degree of the binder is insufficient, resulting in insufficient bonding between the binder and the fiber and other components, so that the impact strength of the friction material is affected and reduced. M2 and B3 have higher impact strength when the hot-pressing temperature is 160 and 165 °C, which are 4.095 kJ/m^3^ and 4.193 kJ/m^3^, respectively. In this case, the bonding strength between the fiber and the matrix is higher. In addition, compared with an ordinary phenolic resin, BPR has a B–O bond, which has better flexibility, thus improving the toughness of the phenolic resin.

Compressive strength is a measure of the ability of a material to resist a compressive load without failure, and its value directly affects the structural integrity and service performance of the material. It is apparent from Table 3 that the compressive strength of MPR-based friction materials decreases with the increase of the hot-pressing temperature, while the compressive strength of BPR-based friction materials increases slightly and then decreases with the increase of the hot-pressing temperature. The main reason is that the hot-pressing temperature affects the curing speed of the resin and the high temperature causes the pressed plastic to harden before it is filled with the mold cavity, resulting in uneven overall bonding. In addition, the variation trend of the compressive strength of friction materials is consistent with that of hardness. Friction materials with greater hardness have a higher compressive strength because the components of friction materials with higher hardness are more closely bonded and can withstand greater pressure without deformation. Figures 3A–F and 4G–L show the compression fracture morphology of all specimens, from which it can be seen that the compression section is unevenly distributed with a significant number of debris still attached to the matrix.

**Figure 3:**
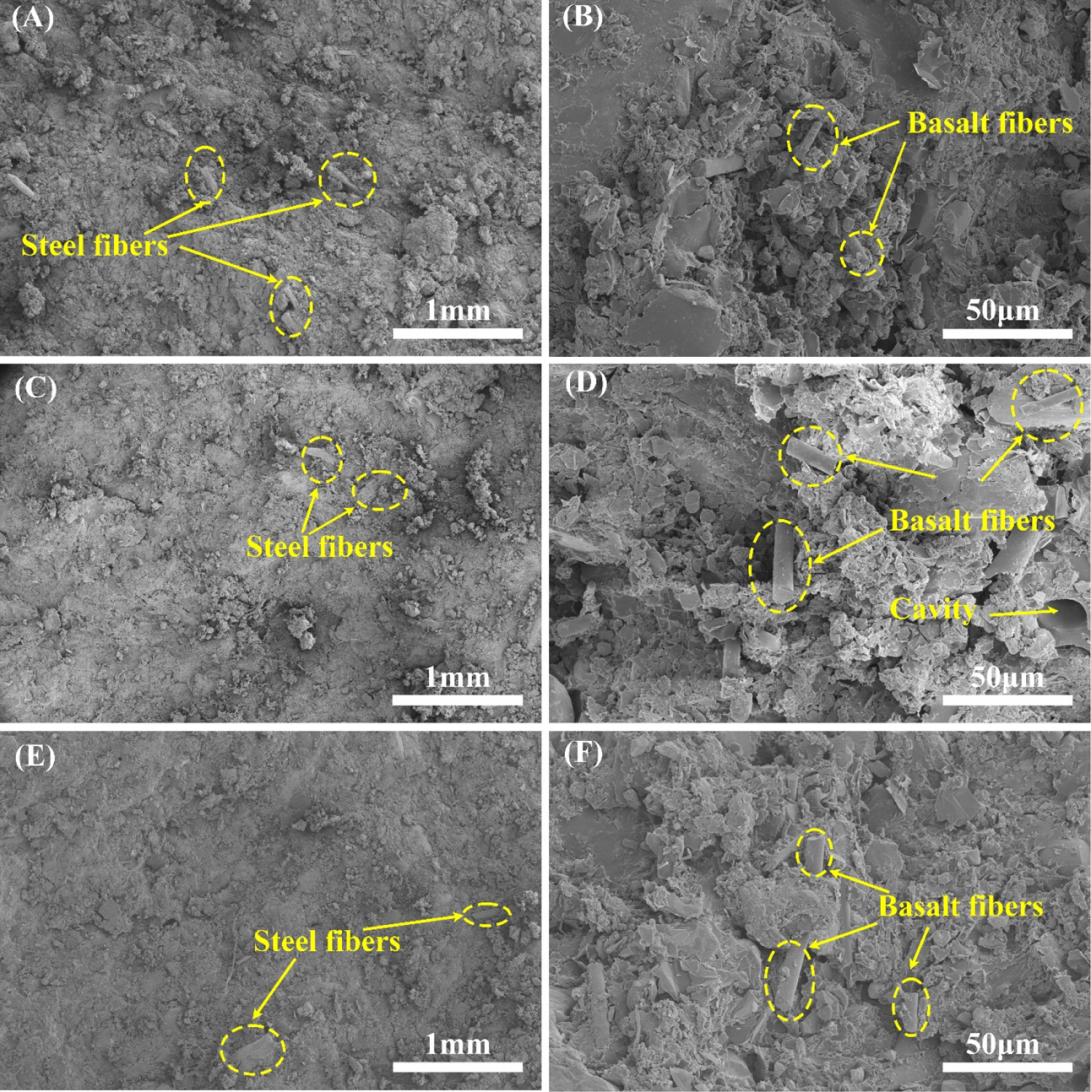
Compression fracture morphology of specimens M1 (A, B), M2 (C, D), and M3 (E, F).

**Figure 4:**
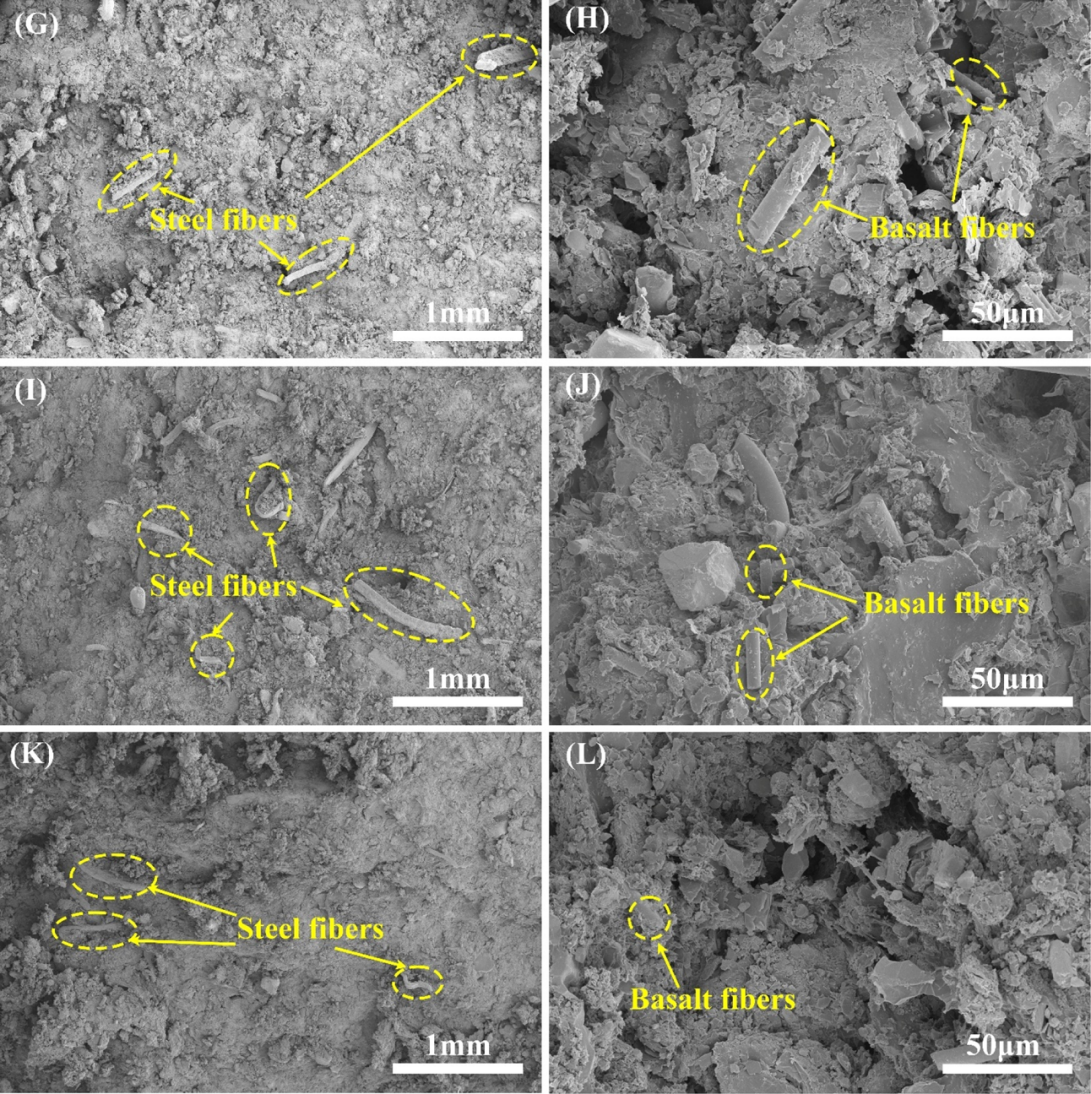
Compression fracture morphology of specimens B1 (G, H), B2 (I, J), and B3 (K, L).

By comparing Figure 3A,C,E, it can be seen that the overall cross section of specimen M3 is flatter than that of specimen M1 and specimen M2, and the number of pulled steel fibers at the cross section of specimen M2 and specimen M3 is also less than that of specimen M1. This indicates that the combination between the components of M3 is weaker than that of M1, and the components are more easily separated, so the plane formed by M3 under compression load is relatively flat. Figure 3B,D,F shows the enlarged images of the compressed fracture morphology of specimens M1, M2, and M3, respectively. Compared with the fracture of specimen M1, the fracture of specimens M2 and M3 has more exposed basalt fibers and debris and more extensive cracks. This further indicates that the bond between the components of specimen M1 is closer. Figure 4G,I,K shows the compression fracture topography of specimens B1, B2, and B3. By comparing the three figures, it is found that the number of exposed steel fibers in the compression section of specimen B2 is more than that of specimen B1 and significantly more than that of B3. At the same time, compared with the enlarged images in Figure 4H,J,L of the compressed fracture morphology of specimens B1, B2, and B3, more voids are distributed in the fracture of specimen B3, which indicates that the combination of components of the B3 sample is relatively loose. This shows that the MPR-based friction materials and BPR-based friction materials have a better hardening rate when the hot-pressing temperature is 155 and 160 °C, respectively, and the cross-linking degree of the pressing plastic is sufficient and uniform.

As shown in Table 3, the compression modulus decreases with the increase of the hot-pressing temperature. Compressive modulus reflects the ability of the material to resist deformation under compressive force, and its size affects the mechanical properties and service life of friction materials. The compression modulus reflects the compressibility of the friction material. The higher the compression modulus is, the smaller the compressibility is. When the compression modulus is too high, the friction material is not easy to deform, which is easy to form a hard point, resulting in the contact between the brake disc and the brake inventory, and the brake disc is stressed unevenly to form local thermal stress. At the same time, too high compression modulus may cause the friction pair to be unable to effectively dissipate heat during friction and cause the temperature to rise.^52,53^

### 3.3 Tribological Properties

#### 3.3.1 Friction Coefficients

The friction coefficient is one of the key parameters used to measure the braking performance of the friction material. Figure 5 demonstrates the average friction coefficient of friction materials with different hot-pressing temperatures at different initial braking speeds. As can be seen from Figure 5, the average friction coefficient of samples M1, M3, B1, and B2 increases first and then decreases with the increase of initial braking speed, which is mainly due to the dense friction film formed by the grinding debris on the friction surface after repeated rolling, thus reducing the friction coefficient, while the average friction coefficient of samples M2 and B3 increases with the increase of the initial braking speed. This may be because, on the one hand, with the progress of braking, the abrasive particles generated by friction are transferred to the friction film to play the role of abrasive particles, which increases the shear strength. At the same time, the hard particles in the abrasive chips are repeatedly squeezed at the interface, which scratch the friction film. On the other hand, according to the friction binomial theorem, the average friction coefficient is proportional to the actual contact area. With the increase of temperature, the decrease of the elastic modulus of the sample leads to the increase of the actual contact area and finally the increase of the friction coefficient.

**Figure 5:**
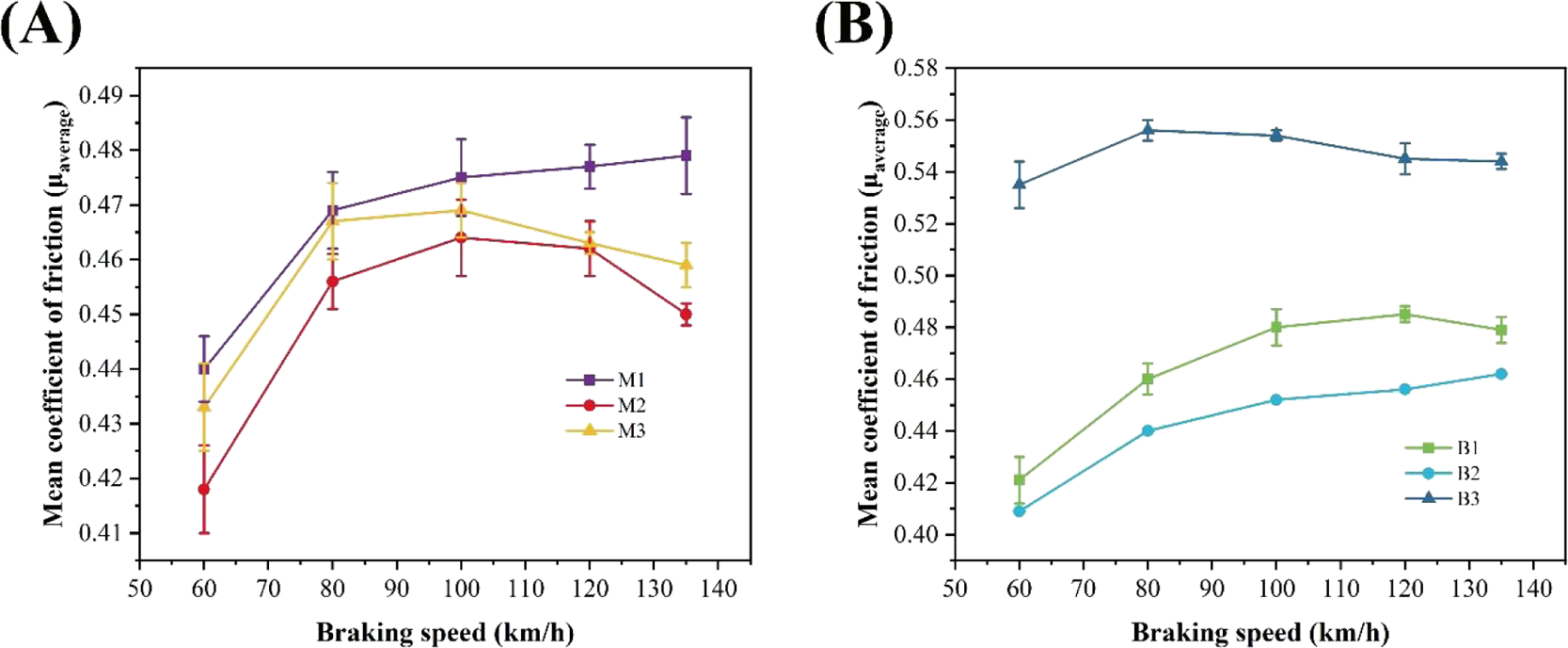
Mean COF versus braking speed for (A) M1, M2, and M3 and (B) B1, B2, and B3.

For MPR-based friction materials, the friction coefficient of specimen M2 with a hot-pressing temperature of 160 °C is higher than that of specimens M1 and M3. The reason may be that specimen M2 has higher impact strength and better toughness; therefore, the actual contact area with the friction pair increases, and the friction coefficient increases accordingly. In addition, as the temperature rises, the combination strength between the components is higher, but the hot-pressing temperature is too high; the hardening rate of the resin is very fast; when the pressed plastic is just poured into the hot-pressing film, there is no time to apply pressure, and part of the resin begins to harden, which will make the pressed plastic well filled with the mold cavity, resulting in the corner defect or texture of the friction product. When friction occurs in the dual parts, the filler and reinforcement fibers that are not well coated cannot provide a good shear force, making the coefficient of friction small. For the BPR-based friction material, the friction coefficient decreases with the increase of the hot-pressing temperature, and the friction coefficient of the B1 specimen is significantly larger than that of B2 and B3 specimens, which probably is because the cross-linking chain of the B1 specimen is denser and the hardening is more sufficient.

The friction curves of the material specimens at different braking speeds are shown in Figure 6A–F. Compared with the friction curves at different braking speeds, the braking time increases with the increase of the initial braking speed. The friction coefficient of different braking speeds soon enters the stable friction stage after a short run-in period. Among them, the friction coefficient of M1 and B2 samples fluctuated most sharply in the run-in stage.

**Figure 6:**
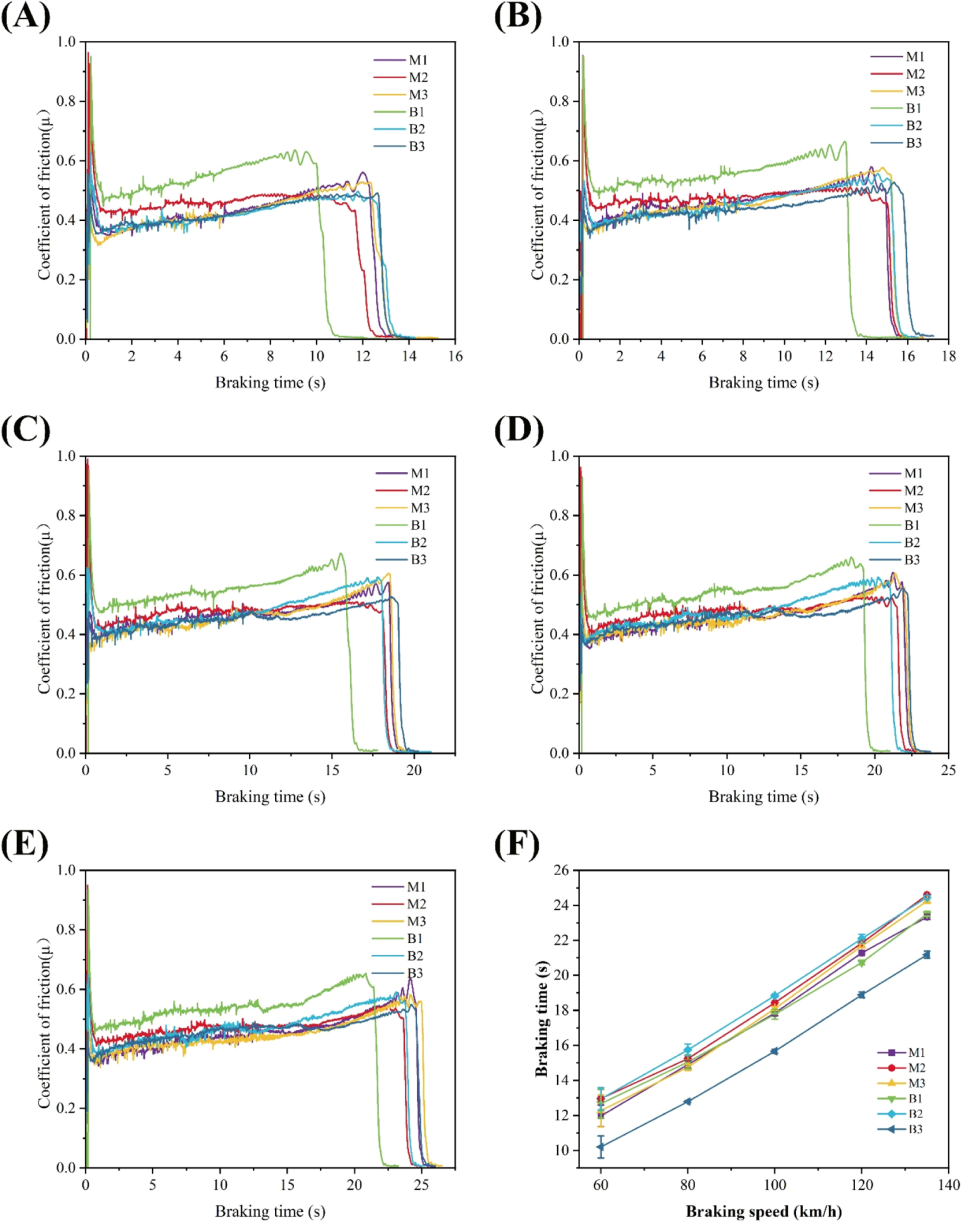
Friction curves for various samples with different braking speeds: (A) 60 km/h, (B) 80 km/h, (C) 100 km/h, (D) 120 km/h, and (E) 135 km/h. (F) The change trend of braking time with the initial braking speed.

Figure 7 shows the relationship between the maximum subsurface temperature and the initial braking velocity of the two resin-based friction materials at different hot-pressing temperatures. The maximum subsurface temperature is the temperature measured 4 mm from the friction surface, and the actual friction surface temperature is much higher than the recorded value. As can be seen from Figure 7, the maximum subsurface temperature of the six friction samples increases with the increase of the initial braking speed. It shows that with the increase of the initial braking speed, the greater the energy required for braking, the more the friction heat will be generated. For the MPR-based friction materials at different hot-pressing temperatures, the subsurface temperature of the samples with larger friction coefficient is higher. For BPR-based friction materials, the maximum subsurface temperature of the B1 specimen is higher than that of the B2 specimen. But the maximum subsurface temperature of the B3 specimen with a small friction coefficient is higher than that of the B2 specimen, and when the braking speed is larger, the maximum subsurface temperature of specimen B3 is also higher than that of specimen B1, which may be because the direct bonding strength of each component of specimen B3 is weak, resulting that friction heat cannot be effectively diffused.

**Figure 7:**
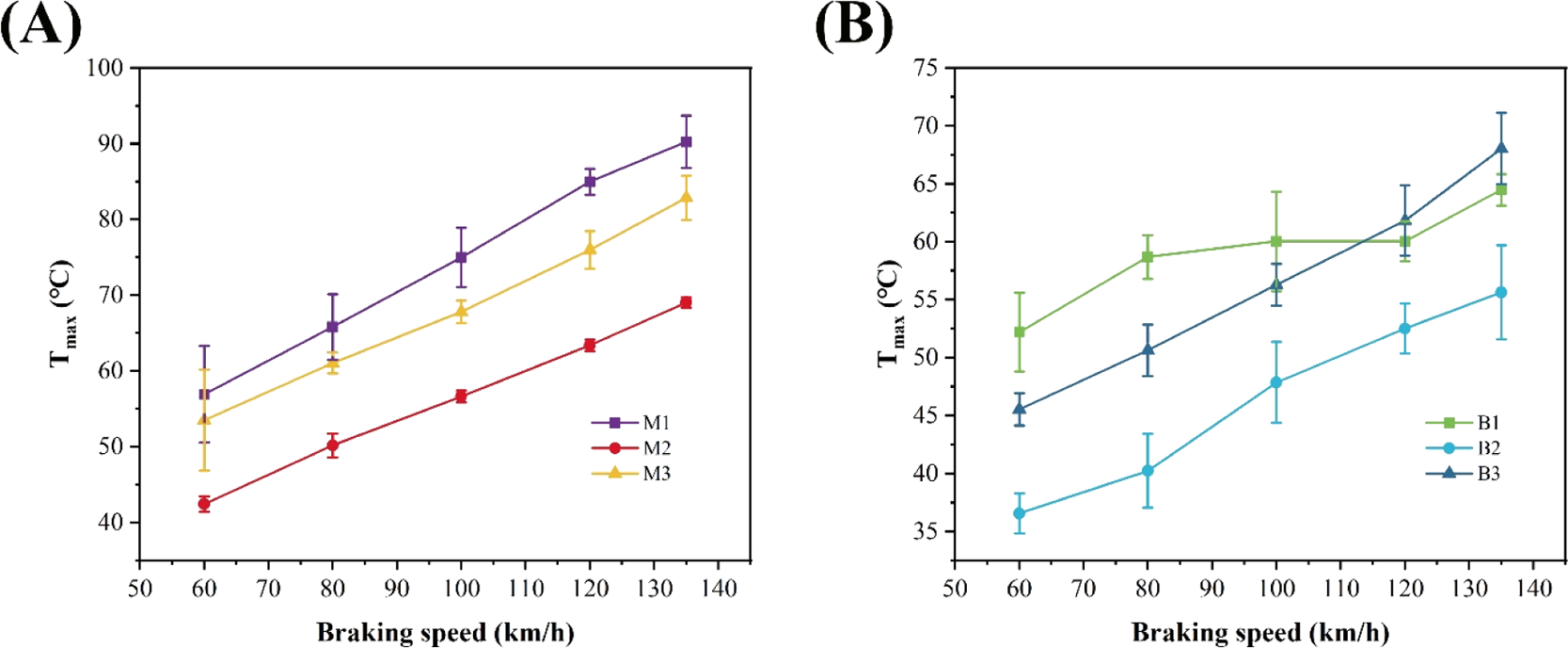
Relationship between the maximum subsurface temperature and braking speed for (A) M1, M2, and M3 and (B) B1, B2, and B3.

The stability of the friction coefficient in the braking process is high, which can reduce the risk of braking loss due to the change of the friction coefficient and reduce the vibration and noise of the vehicle to a certain extent, so as to improve the comfort and safety of the vehicle.^54,55^ The stability of the friction coefficient is characterized by the ratio of the average friction coefficient to the maximum friction coefficient (α). Table 4 shows the friction stability coefficient of each sample at different initial braking speeds. As can be seen from Table 4, the friction stability of specimens M1, M2, and M3 decreased with the increase of the initial braking speed, and the friction stability of specimen M2 was significantly higher than that of specimens M1 and M3, indicating that the friction stability of melamine-modified phenolic resin-based friction materials was better at the hot-pressing temperature of 160 °C. The friction stability of specimen B1 decreases with the increase of the initial braking speed, while that of specimens B2 and B3 first decreases and then increases with the increase of the initial braking speed. The friction stability coefficient of specimen B3 at high speed is the highest compared with that of samples B1 and B2, indicating that the increase in hot-pressing temperature is conducive to the friction stability of boron-modified phenolic resin-based friction materials at high speed.

**Table 4:** Friction Stability Coefficients for Different Samples at Various Braking Speeds

specimens	60 km/h	80 km/h	100 km/h	120 km/h	135 km/h
M1	0.780 ± 0.022	0.805 ± 0.013	0.765 ± 0.017	0.743 ± 0.005	0.705 ± 0.013
M2	0.893 ± 0.021	0.885 ± 0.031	0.878 ± 0.026	0.863 ± 0.026	0.838 ± 0.013
M3	0.790 ± 0.008	0.770 ± 0.012	0.748 ± 0.017	0.738 ± 0.017	0.775 ± 0.013
B1	0.840 ± 0.015	0.845 ± 0.010	0.820 ± 0.006	0.795 ± 0.012	0.805 ± 0.006
B2	0.835 ± 0.006	0.808 ± 0.008	0.798 ± 0.008	0.808 ± 0.010	0.805 ± 0.013
B3	0.835 ± 0.008	0.825 ± 0.010	0.808 ± 0.012	0.813 ± 0.012	0.823 ± 0.011

#### 3.3.2 Specific Wear Rate

The wear scale indicates the wear resistance of friction materials, which directly reflects the length of service life of friction materials in practical applications and is also an important technical and economic index to measure the durability of friction materials. The wear amount of each sample after friction test is shown in Figure 8. With the increase of the hot-pressing temperature, the wear amount of the friction material fluctuates, in which the wear amount of M1 and B2 samples is the smallest and the wear amount of M2 and B3 samples is the largest. The wear of sample B3 is significantly higher than those of samples B1 and B2, which may be because the hardness of sample B3 is too low. When the hardness of the resin-based friction material is too low, the normal load will lead to increased contact area, increased adhesion, and more adhesive wear, resulting in higher wear.

**Figure 8:**
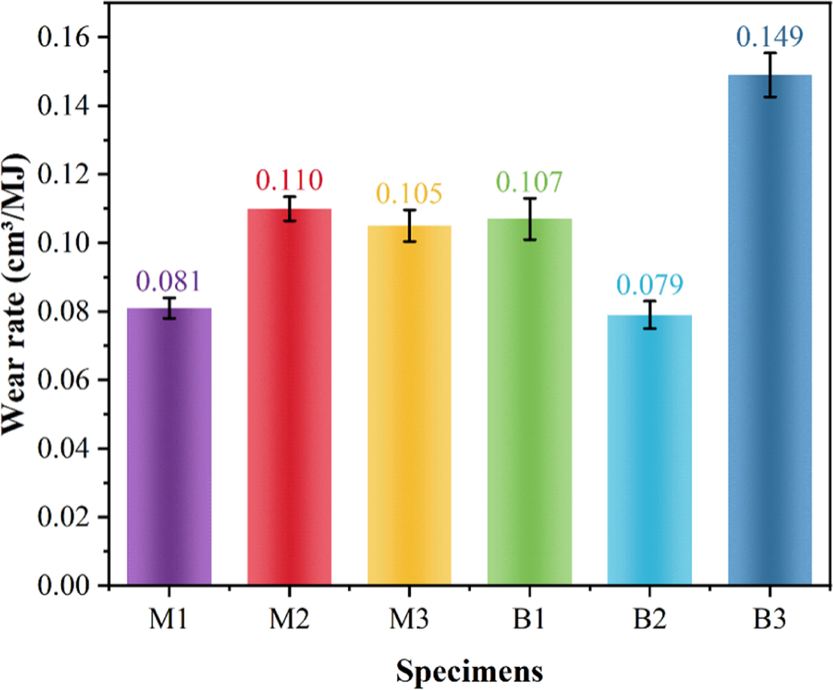
Wear rate of different samples.

### 3.4 Worn Surface Analysis of Friction Materials

In order to further understand the tribological behavior of friction materials, surface electron microscopy (SEM) was used to scan the friction and wear surfaces after braking. Figures 9 and 10 show the wear morphology and the side direction images of the friction film of representative samples M1, M2, B1, and B2 after the whole braking process from low to high speed. As shown in Figure 9A, a large number of discontinuous scale-like friction films are distributed on the wear surface of sample M1, and many deep pits and granular wear chips are also distributed around the friction film. As can be seen from Figure 9B, there are a large number of grooves distributed on the friction film, and the large friction film is less, mainly the stepped and relatively loose scale-like friction film, and there are cracks and a small amount of granular and flaky wear chips around many friction films. In addition, compared with Figure 10I,J, the friction film of the M2 specimen is flatter and denser. The hardness of M1 is high, the cross-linked chain density is high, and the gap between components is small, and there is a large internal stress in the sample matrix, so the matrix is easy to crack and deform during the friction dual process. The wear types of M1 sample are mainly abrasive wear and fatigue wear. Figure 9C shows the wear topography of specimen M2 after the friction test. A small number of pits appear on the wear surface of specimen M2, and a large number of relatively continuous friction films are distributed. As shown in Figure 9D, the friction film area on the wear surface of sample M2 is large and continuous, which is conducive to the stability of the friction coefficient of the friction material. At the same time, many pits are distributed around the friction film, and many fine-grained abrasive chips are accumulated inside the pits. There are a large number of shallow “furrows” on its surface, and a small number of bare fibers that have been compacted by friction are distributed on the wear surface. Therefore, the friction coefficient of the M2 sample is high. The wear mechanism of the M2 specimen is mainly abrasive wear.

**Figure 9:**
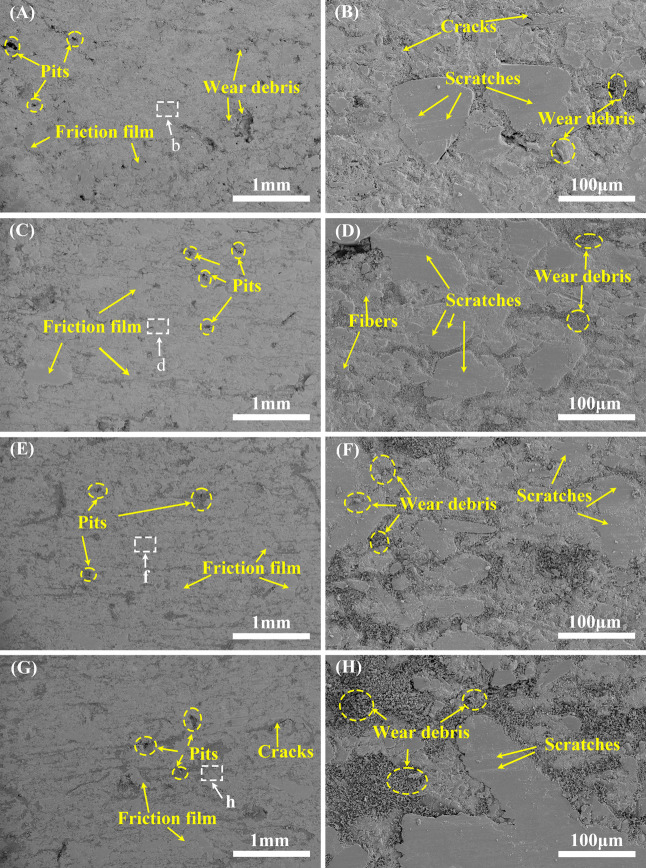
SEM images of the worn surface of specimens. (A) M1 sample, (B) an enlarged view of the rectangular area in (A), (C) M2 sample, (D) an enlarged view of the rectangular area in (C), (E) B1 sample, (F) an enlarged view of the rectangular area in (E), (G) B2 sample, and (H) an enlarged view of the rectangular area in (G).

**Figure 10:**
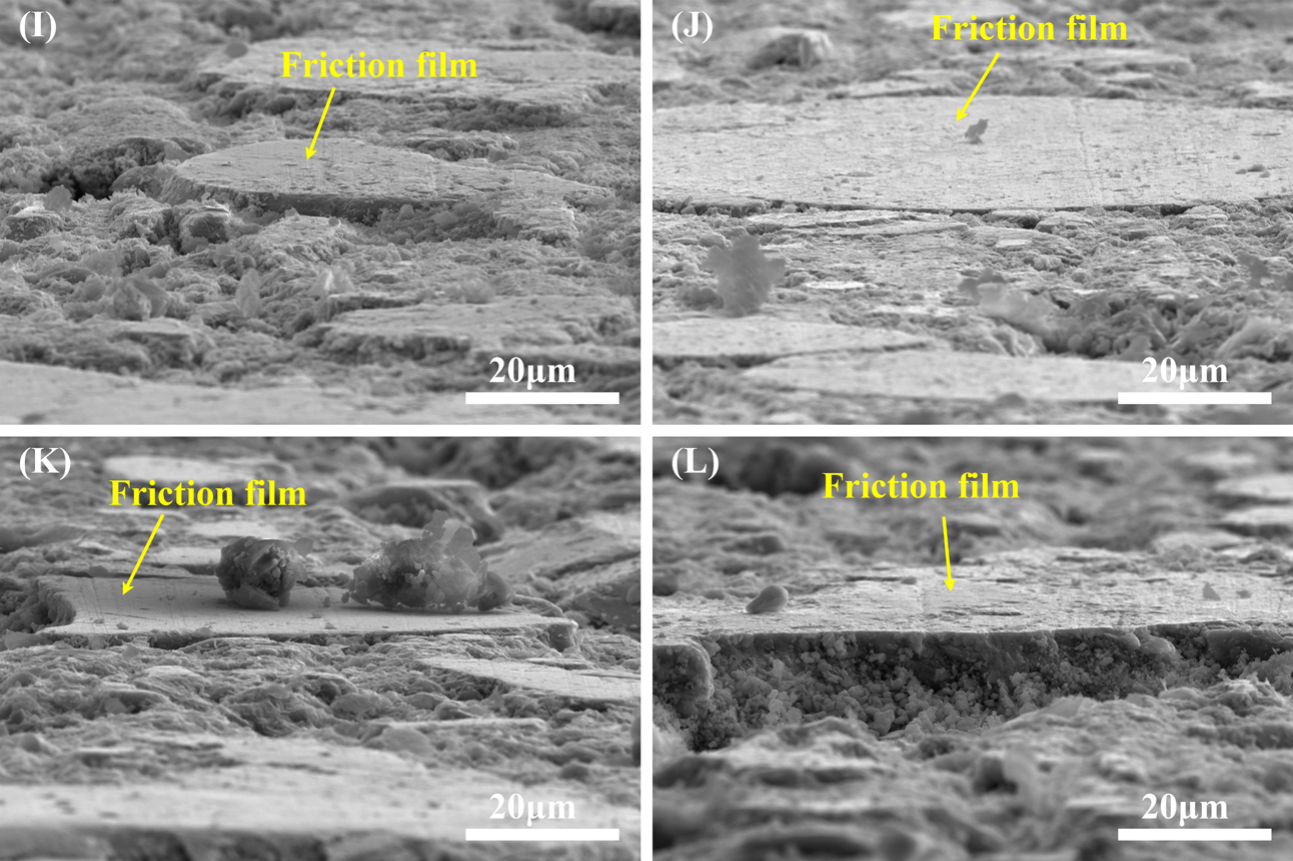
SEM images of the friction film side of specimens. (I) M1 sample, (J) M2 sample, (K) B1 sample, and (L) B2 sample.

The friction coefficient of sample B1 is stable, but the value is high. Combined with Figure 9E,F, it can be seen that there are more complete and smooth film layers distributed on the friction surface. At the same time, a large number of pits are distributed around the friction film, a small number of fine abrasive particles are attached to the surface of the friction film, there are more obvious “furrows” on the surface of the friction film, and a large number of abrasive particles are filled in the pits. Therefore, the average friction coefficient and wear amount of the sample are high, and the wear types are mainly abrasive wear and fatigue wear. It can be observed from Figure 9G that there are only a few discontinuous friction films and many pits of different sizes on the wear surface of sample B2. Combined with the local magnification Figure 9H of the B2 sample, it can be seen that a large number of flacked-up abrasive chips accumulate on and around the friction film, and these abrasive chips form a new and relatively loose friction film after being rolled by friction. It can also be seen from Figure 10K,L that the friction film of the B1 sample is smoother and more abundant. Therefore, the friction coefficient stability of the B2 sample is relatively low, but the wear amount is low. At the same time, there are a small number of “furrows” on the surface of the friction film due to the repeated movement of the particles along the sliding direction after spalling. Therefore, the wear types of B2 specimen are mainly abrasive wear and adhesive wear.

## 4 Conclusion

In this article, the effects of hot-pressing temperature on the mechanical and tribological properties of MPR-based friction materials and BPR-based friction materials were studied. With the increase of the hot-pressing temperature, the hardness and compressive strength of the two friction materials decreased, the friction coefficient also decreased, and the impact strength increased. The compression strength is related to the direct bonding strength of the matrix and of each component. When the hot-pressing temperature is higher than 165 °C, it is not conducive to the combination of the resin and each component, which will lead to the texture of the friction material being loose and the composition being not uniform. The friction test results show that the MPR-based friction materials and BPR-based friction materials have better tribological properties when the hot-pressing temperature is 160 °C. Although the friction coefficient stability of the boron-modified phenolic resin-based friction material at high temperature can be improved by increasing the hot-pressing temperature, the material has better wear resistance and more suitable friction coefficient at 160 °C. The friction film has a positive effect on the stability of the friction coefficient, and the main wear types of modified phenolic resin-based friction materials are abrasive wear, fatigue wear, and adhesive wear.
